# Potential Spermatogenesis Recovery with Bone Marrow Mesenchymal Stem Cells in an Azoospermic Rat Model

**DOI:** 10.3390/ijms150813151

**Published:** 2014-07-24

**Authors:** Deying Zhang, Xing Liu, Jinpu Peng, Dawei He, Tao Lin, Jing Zhu, Xuliang Li, Yuanyuan Zhang, Guanghui Wei

**Affiliations:** 1 Department of Urology, Children’s Hospital of Chongqing Medical University, No.136, Zhongshan 2 RD, Yuzhong District, Chongqing 400014, China; E-Mails: dzdy199@126.com (D.Z.); liux_0217@163.com (X.L.); pink-floyd@foxmail.com(J.P.); dw.he@163.com (D.H.); lintao272@aliyun.com (T.L.); lixuliang2003@aliyun.com (X.L.); 2 Wake Forest Institute for Regenerative Medicine, Wake Forest University School of Medicine, 391 Technology Way, Winston-Salem, NC 27101, USA; 3 Ministry of Education Key Laboratory of Child Development and Disorders, Key Laboratory of Pediatrics in Chongqing, Chongqing International Science and Technology Cooperation Center for Child Development and Disorders, Chongqing 400014, China; E-Mail: jingzhu@cqmu.edu.cn

**Keywords:** mesenchymal stem cell, germ cell, spermatogonial stem cell, transplantation, differentiation

## Abstract

Non-obstructive azoospermia is the most challenging type of male infertility. Stem cell based therapy provides the potential to enhance the recovery of spermatogenesis following cancer therapy. Bone marrow-derived mesenchymal stem cells (BMSCs) possess the potential to differentiate or trans-differentiate into multi-lineage cells, secrete paracrine factors to recruit the resident stem cells to participate in tissue regeneration, or fuse with the local cells in the affected region. In this study, we tested whether spermatogenically-induced BMSCs can restore spermatogenesis after administration of an anticancer drug. Allogeneic BMSCs were co-cultured in conditioned media derived from cultured testicular Sertoli cells *in vitro*, and then induced stem cells were transplanted into the seminiferous tubules of a busulfan-induced azoospermatic rat model for 8 weeks. The *in vitro* induced BMSCs exhibited specific spermatogonic gene and protein markers, and after implantation the donor cells survived and located at the basement membranes of the recipient seminiferous tubules, in accordance with what are considered the unique biological characteristics of spermatogenic stem cells. Molecular markers of spermatogonial stem cells and spermatogonia (Vasa, Stella, SMAD1, Dazl, GCNF, HSP90α, integrinβ1, and c-kit) were expressed in the recipient testis tissue. No tumor mass, immune response, or inflammatory reaction developed. In conclusion, BMSCs might provide the potential to trans-differentiate into spermatogenic-like-cells, enhancing endogenous fertility recovery. The present study indicates that BMSCs might offer alternative treatment for the patients with azoospermatic infertility after cancer chemotherapy.

## 1. Introduction

Stem cell transplantation has become a new therapeutic strategy for restoring organ or tissue structure and function [1,2]. In the treatment of male infertility, spermatogonial stem cell transplantation (SSCT) was first reported by Brinster in 1994 [3] and has since been established as a technological breakthrough in stem cell research and the study of Sertoli cell-germ cell interactions. Autologous, homologous, and exogenous SSCT has been carried out in various species including rodents, bovines, monkeys and even humans [3,4,5,6]. Since spermatogonial stem cells (SSCs) transmit genetic information to their offspring, homologous and exogenous SSCT will inevitably be challenged in terms of reproductive ethics [7].

Genetically-modified somatic cells or stem cells have been intensively investigated [8,9,10,11,12,13,14,15]. Although the induced stem cells express functional genes and act as sperm cells, the risk and safety of using these cells have been of concern. Non-genetically manipulated stem cell therapy might provide a safe, effective solution to male infertility, especially following anticancer chemotherapy [16]. Mesenchymal stem cells (MSCs) from bone marrow or adipose tissues have great potential [17] for tissue repair. MSCs can differentiate into bone, fat, cartilage, muscle, neurons, hepatocytes, insulin-producing cells and skin in the appropriate conditions *in vivo* [1,2,17,18]. Furthermore, bone marrow derived MSCs (BMSCs), which are easy to isolate, have high proliferation rates and have a high potential for differentiation. Based on these characteristics, they may be valuable for use in autologous transplantation. Nayernia *et al.* [19] demonstrated that murine BMSCs are able to differentiate into early germ cells *in vitro* and *in vivo*. Cakici *et al.* [20] recently demonstrated that GFP-traced adipose-tissue-derived mesenchymal stem cells (ASCs) can give rise to sperm-like cells, leading to recovery of fertility in the busulfan-treated azoospermatic rat model. However, in the busulfan-induced azoospermatism model, self-repair of spermatogenesis might not be excluded due to endogenous stem cells. In this pilot study, we tested the role of BMSC in recovery of fertility in azoospermia. We examined the spermatogenic differentiation of BSMC *in vitro* to evaluate the survival and basic biological characteristics of transplanted BMSCs in an azoospermia rat model. We are also investigating sperm cell development *in vivo* in our ongoing study.

## 2. Results

### 2.1. Cell Culture and Labeling

**Figure 1:**
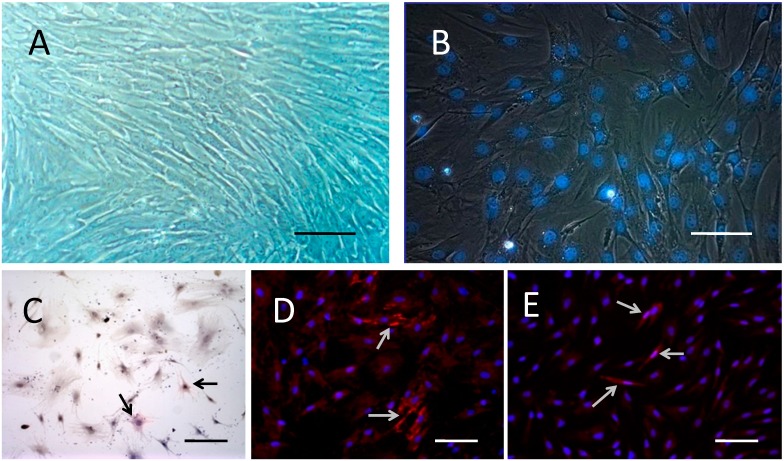
Morphology, labeling and differentiation ability of rat BMSCs. (**A**) BMSCs of passage 4 showed a uniform, long spindle shape, with round or egg-shaped nuclei. The cells were arranged in whirlpool-like shapes, and some cells had double or triple nucleoli; (**B**) After incubation in medium with 10 μg/mL Hoechst33342 for 15 min, the MSC nuclei emitted blue fluorescent light under ultraviolet light; (**C**) Induced by dexamethasone for 1 week, BMSCs manifested as short-fusiform, polygon, irregular scale shaped, with large cell body, and cAKP(+) (arrows); (**D**) Induced by 5-aza for 1 week, some cellular bodies of BMSCs were elongated, branched, uniformly arranged, with some junctions, and cTnT(+) (arrows); (**E**) Induced by salvia miltiorrhiza for 1 day, some BMSCs stretched and connected with each other, shown as Nestin(+) (arrows). Bar: 100 μm.

Primary cultured rat BMSCs began as scattered adherent cells, and grew up into colonies with round nuclei in the middle of the cells. Some cells contained double nuclei. The cells had a high potential of proliferation. After 3–4 passages, the morphology of BMSCs became uniform, with a long spindle shape and round or egg-shaped nuclei. Some cells contained double or triple nucleoli, and cells were arranged in a whirlpool-like shape (Figure 1A). No irregular or pathological mitotic figures were found. The labeled BMSCs pre-induced by retinoic acid and Hoechst 33342 were used for transplantation. After incubation in medium with 10 μg/mL Hoechst 33342, the nuclei appeared blue under ultraviolet light (Figure 1B).

### 2.2. BMSCs Exhibit Multi-Lineage Differentiation Ability

After induction with hexadecadrol for 1 week, the long spindle shaped BMSCs became short-spindled or polygonal in shape, the nucleoplasm ratio increased, and the cells stained positive for AKP (Figure 1C), which correlates with the characteristics of osteoblasts. After incubation with 5-azacytidine, BMSCs were extended with bifurcations and stained positive for cTnT (Figure 1D), demonstrating the features of myocardial cells. However, no cell automatic contraction was observed. After incubation with salvia miltiorrhiza, the cells exhibited features of nerve cells. Double or multi- branches appeared, some of which connected cells. Cells stained positively for nestin (Figure 1E).

### 2.3. Morpholgy Changes and Spermatogenenic Protein Expression of Induced BMSCs in Vitro

**Figure 2:**
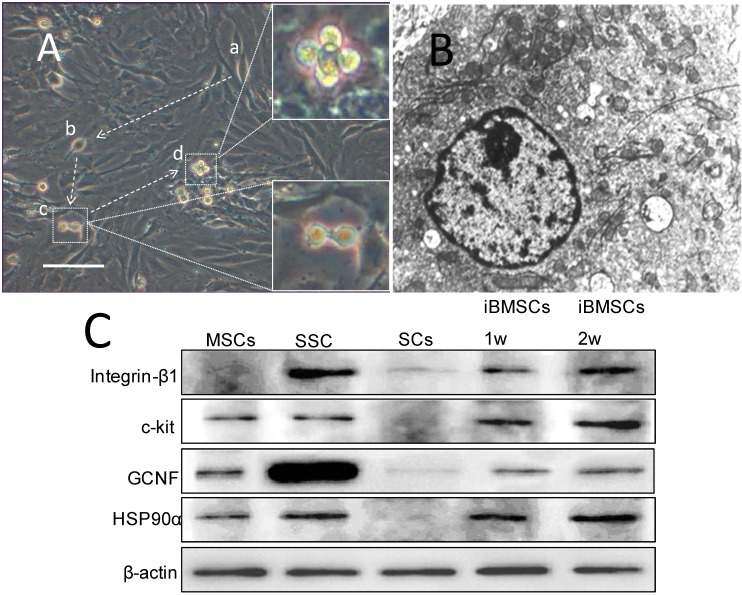
Morphology and biological markers expression by rat BMSCs cultured on the lower layer of the transwell co-culture system in conditioned media. (**A**) Morphology of BMSCs in co-culture system showed small round cells, and double round cells with intercellular bridges. Allied cells were observed. (**a**,**b**) Cells became smaller and rounder in shape; (**c**) right lower insert: double-round-cells, with intercellular bridge; (**d**) right upper insert: allied cells; arrow: the presumed dynamic morphology changes (bar: 100 μm); (**B**) Transmission electron microscope examination showed that BMSCs in the co-culture system exhibited large round nuclei, obvious chromatospherites, more heterochromatin in the nucleus, and more mitochondria in intracytoplasm (×8000); (**C**) Expression of spermatogonial specific protein in each group of cells detected by Western blot: induced BMSCs expressed integrin-β, which was not expressed in BMSCs, expressed higher levels of c-kit and GCNF than that in non-induced BMSCs.

In the *in vitro* study, the BMSCs on the upper layer of the co-culture system showed specific morphologic changes. On the 3rd day of co-culture, some of the cells became smaller and rounder in shape, with higher refractivity. On the 5th day, double-round-cells appeared with an intercellular bridge. On the 7th day, highly refractive cells increased, and allied cells appeared (Figure 2A). The round cells adhered to the wall of the flask, whereas the dead cells floated. The cells exhibited large round nulei, obvious chromatospherites, increased heterochromatin in the nucleus, and many mitochondria in the intracytoplasm under transmission electron microscopy (Figure 2B). Spermatogenic cell markers were detected by western blot, and it was shown that BMSCs co-cultured with Sertoli cells in conditioned media expressed integrin-β, which was not expressed in BMSCs, and the co-cultured cells expressed higher levels of c-kit and germ cell nuclear factor (GCNF) than BMSCs (Figure 2C).

### 2.4. Recipient Rats

**Figure 3:**
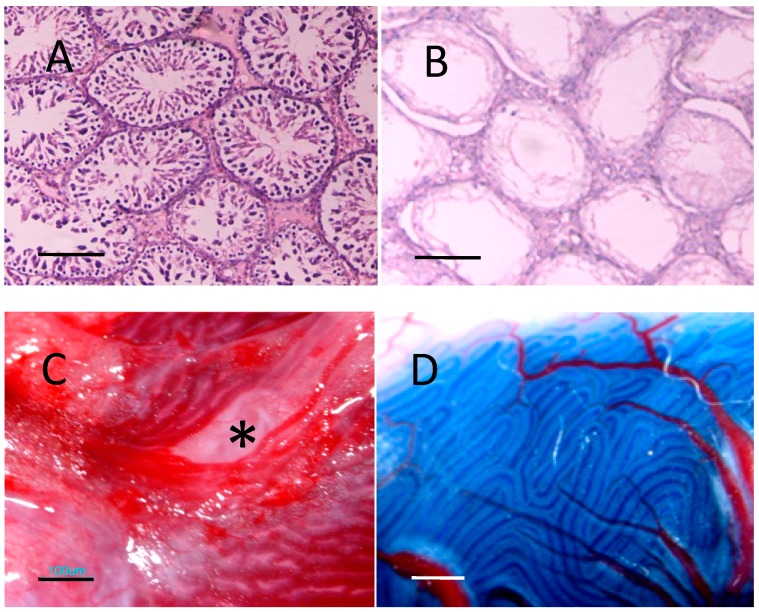
Busulfan induced azoospermatism model preparation and BMSCs transplantation via rete testis. (**A**) Histological morphology of untreated testicle, as normal control (bar: 50 μm); (**B**) Histological morphology of testicular tissue 1 month after injection with 20 mg/kg busulfan. Most of the endogenous sperm cells had been removed, while interstitial tissue and Sertoli cells remained; some seminiferous tubules were visible as Sertoli-cell-only structures (bar: 50 μm); (**C**) Rat rete testes under dissecting microscope, asterisk: site of injection (bar: 100 μm); (**D**) Transplantation of donor BMSCs suspension containing trypan blue into seminiferous tubules by rete testes microinjection, trypan blue traced donor cell suspension injected into recipient seminiferous tubules (bar: 100 μm).

Four weeks after the injection of rats with busulfan, most of the endogenous sperm cells were removed while the interstitial tissue and Sertoli cells remained; some seminiferous tubules appeared as Sertoli-cell-only structures (Figure 3B). After injection, the trypan blue marked BMSCs donor cell suspension filled to more than half the surface seminiferous tubules (Figure 3C,D). Up to 8 weeks after transplantation, no recipient rats died, no swelling, lymphoid cells or granular leukocyte aggregations were found in the recipient testicular tissue, and there was no evident tumor mass anywhere in the body.

### 2.5. Donor Cells in Recipient Seminiferous Tubules

**Figure 4:**
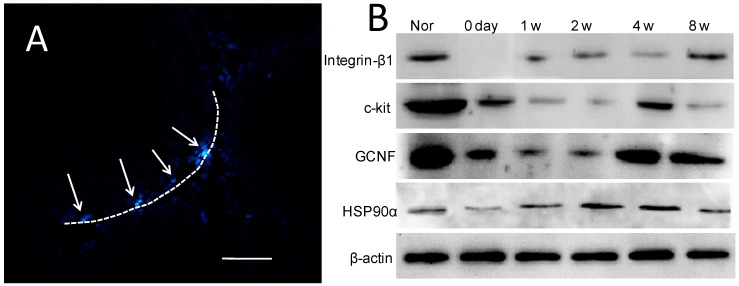
BMSCs location post-transplantation and spermatogenesis marker expression in recipient testes: (**A**) A few donor BMSCs labeled with Hoechst 33342 were detected in the basement membranes of the seminiferous tubules 1 week after transplantation (arrow), dotted line indicates basement membrane of seminiferous tubule (bar: 20 μm); (**B**) Western blots showing expression of spermatogenic cell-specific proteins in recipient testicular tissue after BMSCs transplantation.

One week after transplantation, most of the donor cells stained with Hoechest 33342 had been eliminated from the recipient testes; only a few remained on the basement membranes of the recipient seminiferous tubules (Figure 4A).

### 2.6. Expression of Spermatogenic Molecular Markers in Recipient Testicular Tissue

**Figure 5:**
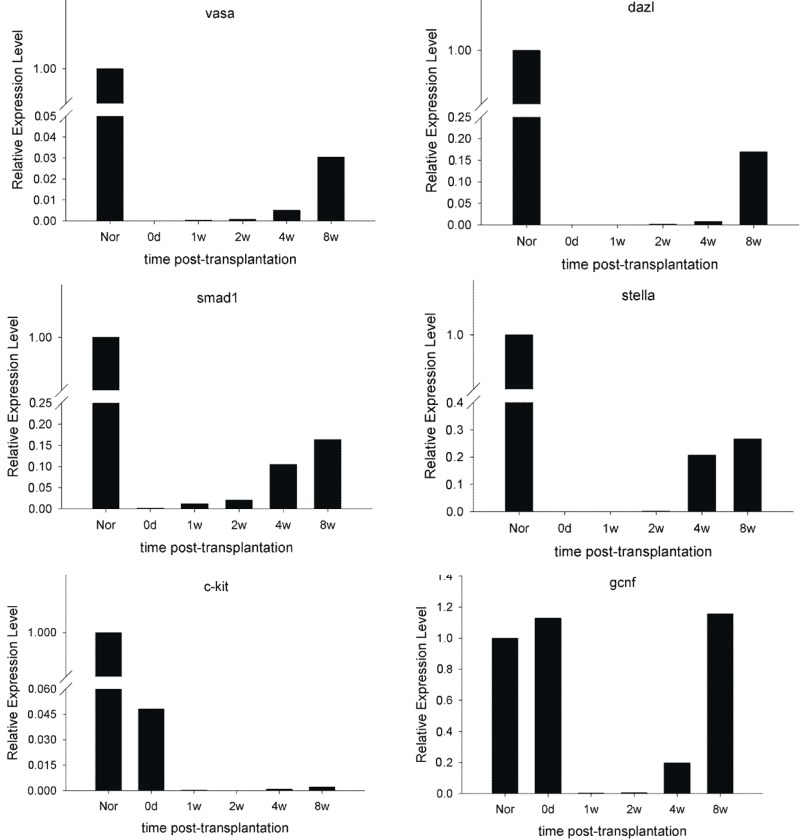
Relative level of spermatogenic specific genes expression in recipient rat testicular tissue after BMSCs transplantation detected by real time PCR. Vasa, dazl, smad1 and stella expression were detected 2 weeks post-transplantation and increased to a relatively high level at 4 to 8 weeks. C-kit and GCNF were expressed at relatively high levels immediately after injection, and expression levels decreased to almost zero within 2 weeks. GCNF expression increased gradually at 4 weeks post-transplantation, while expression of c-kit remained at a relatively low level.

The known molecular markers of spermatogonial stem cells and spermatogonia—*Vasa*; *Dazl*; *Stella*; *SMAD1*; *GCNF*; *c-kit* mRNAs; and integrinβ1; HSP90α; GCNF; c-kit proteins—were detected in the recipient testes 1–8 weeks after transplantation (Figure 4B and Figure 5; Table 1). GCNF and c-kit expression was detected immediately after injection; decreased 1 week later; recovered after 2 weeks; and gradually increased thereafter. Expression of Vasa; Dazl; Stella; SMAD1; Oct-4; integrinβ1 and HSP90α increased gradually between 1 and 8 weeks post-transplantation. There was no evidence that the donor cells underwent meiotic differentiation.

**Table 1:** Expression of spermatogenic cell-specific proteins after MSC transplantation.

Group	Integrin-β1	c-kit	GCNF	HSP90α
Nor	0.77 ± 0.10	2.17 ± 0.50	1.84 ± 0.70	0.65 ± 0.17
0 day	0.01 ± 0.01 *	0.94 ± 0.19 *	0.48 ± 0.13 *	0.21 ± 0.08 *
1 week	0.11± 0.05 *	0.62 ± 0.17 *	0.27 ± 0.09 *	0.45 ± 0.06 *
2 weeks	0.20 ± 0.08 *	0.27 ± 0.08 *	0.35 ± 0.09 *	0.65 ± 0.13
4 weeks	0.33 ± 0.12 *	0.85 ± 0.14 *	1.30 ± 0.23	0.76 ± 0.09
8 weeks	0.69 ± 0.22	0.75 ± 0.24 *	1.36 ± 0.25	0.71 ± 0.11

* compared with Nor group, *p* < 0.05.

## 3. Discussion

Approximately 1% of all men in the general population suffer from azoospermia, obstructive or non-obstructive, and azoospermic men constitute approximately 10%~15% of all infertile men [21]. Among this population, men with non-obstructive azoospermia (NOA) are the most difficult to treat. Various conditions can cause NOA, including genetic or congenital abnormalities, infectious issues, exposure to gonadotoxins, medications such as chemotherapy reagents, varicocele, trauma, endocrine disorders, and idiopathic causes. Current medical therapy, including hormone or surgical methods, shows little benefit to NOA [22,23].

Recent regenerative medical research has shed light on this problem. Human somatic stem cells could be induced to differentiate into multi-lineage cells that help to replace and rebuild damaged or mis-functioning tissues and organs [16]. Among the stem cells used in regenerative medicine, BMSCs are easily collected, have a high proliferation and differentiation potential, and have low immunological suppression and rejection, therefore BMSCs are suitable for autologous stem cell therapy [17].

The particular microenvironments in which BMSCs are implanted plays a vital role in the course of their differentiation [2,24,25]. Seminiferous tubules physically provide dynamic and cyclic regulation of spermatogenesis, and testicular Sertoli cells form a microenvironment that is conducive to sperm cell differentiation and proliferation. In this study, rat BMSCs which were co-cultured with Sertoli cells in a transwell system and in conditioned media *in vitro*, were transplanted into the seminiferous tubules of busulfan-treated infertile rats *in vivo*. This provided an appropriate spermatogenic microenvironment (niche) to investigate whether they could transdifferentiate into sperm cells. Because cells in normal seminiferous tubules are hierarchically organized with regard to spermatogenesis [26], endogenous germ cells must be removed so that spermatogonial stem cell niches are opened and rendered accessible to the transplanted donor cells [27,28]. Busulfan-treated rats are excellent and well-established recipients for evaluating stem cell activity in rat testis cell populations [27]. They were used as recipients in this study. Having vacant stem cell niches, favorable growth factors/hormonal milieu, and no Sertoli cell tight junctions provide a suitable physical microenvironment for spermatogenesis.

In this study, rat BMSCs were co-cultured with Sertoli cells in conditioned media *in vitro*, which mimics the spermatogenic microenvironment. The BMSCs became round and allied, and exhibited specific morphological characteristics of spermatogonia. After induction, BMSCs expressed integrin-β. Both the post-induced and pre-induced BMSCs expressed c-kit and GCNF, although the former exhibited these markers at higher levels. This suggests that because BMSCs and sperm cells share some markers, BMSCs have the potential to transform into sperm cells. Although Lassalle *et al.* [29] reported that mouse BMSCs could not be transformed into sperm cells *in vivo*, we found that rat BMSCs pre-induced with vitamin A *in vitro* can survive in recipient rat testes *in vivo*, migrate, and become implanted in the basement membranes of seminiferous tubules, considered the unique biological characteristics of spermatogenic stem cells [26]. Furthermore, the molecular markers of spermatogonial stem cells and spermatogonia—Vasa, Stella, SMAD1, Dazl, GCNF, HSP90α, integrinβ1 and c-kit—were expressed in the recipient testicular tissue after transplantation, indicating that BMSCs transplanted into seminiferous tube probably transdifferentiate into spermatogenic cells. Only a few of the numerous donor BMSCs survived in the recipient and became implanted in the basement membranes of the seminiferous tubules, which indicates that only a small portion of BMSCs have the potential to differentiate into germ cells. Up to 8 weeks after transplantation, no meiosis was found. The reason for this arrest of differentiation is still unknown; therefore, long-term observation must be continued.

Another feature of BMSCs is that they are not only hypo-immunogenic but also produce immunosuppression or immunosurveillance upon transplantation, so they are suitable for allogeneic transplantation [24,30,31]. Also, Sertoli cells are immune tolerant cells [32]. This ultimately benefits the survival of the donor BMSCs in the recipient seminiferous tubules, so it is not surprising that no immune or inflammatory reaction occurred post transplantation. Although no tumor mass was found 8 weeks post-injection in the recipient rats, the safety and tumorigenic potential of BMSC transplantation still requires long-term observation.

Several previous studies demonstrated that mesenchymal stem cell transplantation recovered fertility in busulfan-treated azoospermatic rats [19,20,33,34]. Although the results were encouraging, the mechanism is unclear. There are three possibilities for MSCs to recover cell or tissue function during the tissue regeneration process: (1) MSCs differentiated into the target function cells via appropriate induction conditions [1,17,18]; (2) Stem cells secreted trophic factors to stimulate the endogenous stem cells or restore the injured host cell function [2]; or (3) MSCs merge with the resident cells to recover the injured cell function [35,36]. In this study, we demonstrated that spermatogenically-differentiated BMSCs expressed integrin-β, c-kit, and GCNF *in vitro* and located at the basement of seminiferous tubule after implanted. However, the role of induced BMSCs *in vivo* is still unclear. Further experiments are needed to determine the effect of trans-differentiation, trophic effect or cell fusion of BMSCs on spermatogenesis. Bhartiya *et al.* [37,38,39] recently reported that there is a small population of pluripotent stem cells, described as “very small embryonic-like stem cells (VSELs)”, which exists in various adult body tissues, including bone marrow. In our experiment, BMSCs may have been contaminated with VSELs; This explains the following: (1) only a small amount of BMSCs exhibited the morphology of spermatogenic stem cells when co-cultured with Sertoli cells *in vitro*; (2) only a small amount of BMSCs survived and located in the basement of the seminiferous tubule in the azoospermatic rat model after transplantation; (3) the possibility of MSCs, the mesoderm originated cells can give rise to sperm cells, the endoderm lineage cells, without genetic modifications.

There are some limitations to this study. Long term observation is difficult without a long term traceable labeling method, and without a negative control group (injection with only medium and trypan blue without cells), the possibility of endogenous recovery of spermatogenesis in the busulfan-induced azoospermic model cannot be excluded. Further experiments on genetic traceable GFP+ cell labeling, appropriate control groups, and long-term follow up will help better understand the spermatogenesis support of BMSCs.

## 4. Materials and Methods

### 4.1. Experimental Animals

Male Sprague-Dawley (SD) rats of 4–6 weeks old were obtained from the Experimental Animal Center, Chongqing Medical University, Chongqing, China. The Animal Ethical Committees of the Institute of Zoology approved the use of animals for the study.

### 4.2. BMSCs Collection, Culture and Differentiation Potential Test

BMSCs were isolated from the rats following the method described by Gnecchi *et al.* [17,40] and Pereira *et al.* [40]. Cells were cultured in Dulbecco’s Modified Eagle’s Medium/nutrient mixture F12 Ham medium (DMEM/F12 1:1, Gibco, New York, NY, USA) with 10% FBS, 37 °C, 5% CO_2_, and passaged when they reached 80%–90% confluence. To test the differentiation ability, cells were subcultured on poly-l-lysine coated slides in 24-well plates, 10 nmol/L hexadecadrol, 10 μmol/L 5-azacytidine, 100 g/L Salvia miltiorrhiza, and were incubated for 1 week, 1 week, and 1 day respectively. The osteoblastic marker cAKP was detected by the modified Kaplow method. Myocardial cell marker cTnT and nerve cell marker nestin were detected by immunocytochemistry according to the instructions.

### 4.3. Induction of BMSCs to Spermatogenic Differentiation in Vitro by Co-Culture with Testicular Sertoli Cells in Conditioned Media

The MSCs of passage 4 were used for spermatogenic differentiation induction *in vitro*. Primary testicular Sertoli cells were isolated from 0 to 1 day old rats following the method described by Grima *et al.* [41], and cultured on the 6-well plates of the upper layer of transwell co-culture system (Corning Inc., New York, NY, USA). The Sertoli cells were treated with mitomycin C (10 μg/mL in DMEM/F12) for 3 h to restrict further proliferation when they reached 80%~90% confluence. BMSCs were seeded on the transwell membrane of the lower layer of co-culture system. Conditioned media (DMEM/F12 medium with 10% FBS, 1 mmol/L sodium pyruvate, non-essential amino acid, minimum essential vitamin, 0.1 mmol/L β-mercaptoethanol (Invitrogen, Carlsbad, NM, USA), and 0.5 mmol/L LIF (Sigma, St. Louis, MI, USA), was added to the co-cultured cells which were cultured in a 34 °C incubation room with 5% CO_2_ and 95% humidity. Morphology of BMSCs was observed under inverted microscopy and transmission electron microscopy, and the spermatogenic specific proteins were detected by western blot.

### 4.4. Preparation of Donor MSCs for Transplantation in Vivo

The BMSCs of passage 4 were incubated in DMEM/F12 medium with 10% FBS and 20 μmol/L retinoic acid (Sigma, St. Louis, MI, USA) for 3 days before transplantation. They were then incubated in 10 mL medium containing 10 μg/mL Hoechst 33342 (Promega, Madison, WI, USA) for 15 min, washed at least three times with PBS and trypsinized. The final cell suspension (10^6^ cells/mL) in FBS-free DMEM/F12 with 10% (*v*/*v*) trypan blue was ready for transplantation.

### 4.5. Preparation of Recipient Rats: Busulfan-Induced Azoospermatism Model

The busulfan-treated infertile rat model was prepared as described by Brinster *et al.* [27]. SD rats were used as recipients 4 weeks after single dose intraperitoneal injection with busulfan (40 mg/kg, Sigma, St. Louis, MI, USA) at 4 weeks of age. Hematoxylin-Eosin stain of testicular cross section was performed to evaluate the recipient model 4 weeks after busulfan injection.

### 4.6. BMSCs Transplantation and Testicular Tissue Collection

The donor BMSCs suspended in serum-free DMEM/F12 were injected into the seminiferous tubules of the recipient rats, with introduction into the rete (is this supposed to be rat?) testes, as described by Brinster and Ogawa [3,42]. Approximate 100 μL (10^5^ cells) of BMSCs suspension was introduced into the tubules in the recipient testis, which filled more than half the surface seminiferous tubules. The recipient rats were anesthetized with chloral hydrate injection (30 mg/kg, i.p.) for transplantation. The testes of the recipient rats were collected and fixed in 10% neutral buffered formalin or kept in −80 °C for paraffin sections and spermatogenic markers were detected immediately after the injection at 1, 2, 3, 4 and 8 weeks later.

### 4.7. RNA Extraction and Quantitative Real-Time RT-PCR

Total RNA was extracted from the testis tissue using an RNApure total RNA isolation kit (Bioteke, Beijing, China) according to the manufacturer’s instructions. First-strand cDNA was obtained from 5 μL of the total RNA using an AMV First Strand cDNA Synthesis Kit (QiaGEN, Shanghai, China). Amplification reactions were performed in a total volume of 25 μL of PCR mixture from the SYBR Green I Real Time PCR KIT (Takara, Beijing, China) containing 5 μL 5× PrimeSTAR™ buffer (Mg^2+^ plus), 2 μL dNTPs (2.5 mM each), 0.25 μL PrimeSTAR™ HS DNA polymerase (2.5 U/μL), 0.5 μL first-strand cDNA, 0.5 μL (20 pmol) each of the specific primers for *Vasa*, *SMAD1*, *Stella*, *Dazl*, *GCNF*, *c-kit*, and *β-actin* (as reference) (for primer sequences see Table 2), and 16.25 μL RNase-free water. The samples were denatured at 94 °C for 3 min, followed by 40 amplification cycles of 94 °C for 30 s, 50, 51, 56, 58, 55, 58 and 56 °C (for *Vasa*, *SMAD1*, *Stella*, *Dazl*, *GCNF*, *c-kit*, and *β-actin* respectively) for 15 s, and 72 °C for 30 s, in a thermal cycler (Roche, Basel, Switzerland); fluorescence signal intensity was measured at 72 °C during each cycle. The PCR products were identified by melting curves: 95 °C for 2 min, 72 °C for 1 min, 95 °C for 30 s with steps of 0.5 °C/s, 30 °C for 1 min. Each product represented a single peak.

### 4.8. SDS-PAGE and Western Blotting

Proteins were extracted from the cells or testis tissue using Radioimmunoprecipitation assay (RIPA) buffer (Sangon, Shanghai, China) containing 1 mM phenylmethylsulfonylfluoride (PMSF). SSCs and testicular tissue from normal 6-week-old male rats were used as a positive control. Each protein sample (20 μL) was separated on a 12% SDS-polyacrylamide gel (Invitrogen, Carlsbad, NM, USA) and blotted on to a polyvinylidene fluoride (PVDF) membrane (Bio-Rad, Shanghai, China). Protein binding sites were blocked for 1~2 h with blocking buffer: TBST (Tris-buffered saline with Tween-20: 10 mM Tris–HCl, pH 7.5, 150 mM NaCl, 0.05% Tween-20) containing 5% nonfat dry milk at room temperature (RT). Primary antibodies were diluted in blocking buffer and incubated overnight at 4 °C: GCNF (1:600), HSP90α, integrinβ1, c-kit (1:300), β-actin (1:3000). The membranes were washed three times with TBST for 10 min. After incubation with secondary antibodies: goat-anti-rabbit-HRP or rabbit-anti-mouse-HRP (1:5000, Santa Cruz, Dallas, TX, USA) in blocking buffer for 1 h at RT, the membranes were again washed three times in TBST for 10 min. Protein bands were detected by the enhanced chemiluminescence method (ECL, QiaGEN, Shanghai, China). The area and density of each protein band were measured and the relative level of target protein was evaluated by the ratio of its area density to that of β-actin.

**Table 2:** Primer sets used for quantitative real-time PCR.

Target Gene	Locus No.	Sequence of Primers	Product Size
*Vasa*	S75275	F: 5'-GCGAGACTACATCTACAAC-3'	135 bp
		R: 5'-GAGTATCTTCACAGTCATTA-3'	
*SMAD1*	AF067727	F: 5'-CTCATGTCATTTATTGCCG-3'	138 bp
		R: 5'-CTCGCTTATAGTGGTAGGGA-3'	
*Stella*	BK001414	F: 5'-CTATCATCGTCGTCAAAGG-3'	177 bp
		R: 5'-CTCTGCTCAATCCGAACAA-3'	
*Dazl*	NM_001025742	F: 5'-CGACGAAATCGGGAAGCTC-3'	94 bp
		R: 5'-CACAACCTCACCATACTGGGAAA-3'	
*GCNF*	AJ783965	F: 5'-CAACTGAACAAGCGGTATT-3'	114 bp
		R: 5'-GATGTATCGGATCTCTGGC-3'	
*c-kit*	NM_022264	F: 5'-TGCCCGAAACAAGTCATCTCC-3'	112 bp
		R: 5'-GGCTGAGGGTTCAACTTTATCCA-3'	
*β-actin*	NM_031144	F: 5'-GCTCGTCGTCGACAACGGCTC-3'	353 bp
		R: 5'-CAAACATGATCTGGGTCATCTTCTC-3'	

### 4.9. Statistical Analysis

Experiments were repeated at least three times. qPCR results were expressed by *C*_t_ values and calculated as follows: Δ*C*_t_ = *C*_t(target gene_) − *C*_t(β-actin)_; ΔΔ*C*_t_ = Δ*C*_t(transplantation group)_ − Δ*C*_t(control group)_. The relative levels of target gene expression were evaluated as 2^−ΔΔ*C*t^. Relative protein levels were expressed as means ± SEM and analyzed by the Student-Newman-Keuls test using SPSS 18.0. A value of *p* < 0.05 was chosen as an indication of statistical significance.

## 5. Conclusions

The spermatogenetic process of the grafted stem cell includes three main steps: *in vitro* induction, cell transplantation, and sperm cell development *in vivo*. This study demonstrated that induced BMSCs express spermatogenesis-related genes and protein markers *in vitro* and homed to the basement of seminal seminiferous tubule. This warrants further investigation on germ cell development of BMSCs, providing potential treatment for patients with azoospermatism, especially for those undergoing chemotherapy.
